# Research on herd sheep facial recognition based on multi-dimensional feature information fusion technology in complex environment

**DOI:** 10.3389/fvets.2025.1404564

**Published:** 2025-03-13

**Authors:** Fu Zhang, Xiaopeng Zhao, Shunqing Wang, Yubo Qiu, Sanling Fu, Yakun Zhang

**Affiliations:** ^1^College of Agricultural Equipment Engineering, Henan University of Science and Technology, Luoyang, China; ^2^Collaborative Innovation Center of Machinery Equipment Advanced Manufacturing of Henan Province, Luoyang, China; ^3^College of Physical Engineering, Henan University of Science and Technology, Luoyang, China

**Keywords:** YOLOv5, MCFB, SSSA, compression of model, transfer learning

## Abstract

Intelligent management of large-scale farms necessitates efficient monitoring of individual livestock. To address this need, a three-phase intelligent monitoring system based on deep learning was designed, integrating a multi-part detection network for flock inventory counting, a facial classification model for facial identity recognition, and a facial expression analysis network for health assessment. For multi-part detection network, The YOLOv5s path aggregation network was modified by incorporating a multi-link convolution fusion block (MCFB) to enhance fine-grained feature extraction across objects of different sizes. To improve the detection of dense small targets, a Re-Parameterizable Convolution (RepConv) structure was introduced into the YOLOv5s head. For facial identity recognition, the sixth-stage structure in GhostNet was replaced with a four-layer spatially separable self-attention mechanism (SSSA) to strengthen key feature extraction. Additionally, model compression techniques were applied to optimize the facial expression analysis network for improved efficiency. A transfer learning strategy was employed for weight pre-training, and performance was evaluated using FPS, model weight, mean average precision (mAP), and test set accuracy. Experimental results demonstrated that the enhanced multi-part identification network effectively extracted features from different regions of the sheep flock, achieving an average detection accuracy of 95.84%, with a 2.55% improvement in mAP compared to YOLOv5s. The improved facial classification network achieved a test set accuracy of 98.9%, surpassing GhostNet by 3.1%. Additionally, the facial expression analysis network attained a test set accuracy of 99.2%, representing a 3.6% increase compared to EfficientNet. The proposed system significantly enhances the accuracy and efficiency of sheep flock monitoring by integrating advanced feature extraction and model optimization techniques. The improvements in facial classification and expression analysis further enable real-time health monitoring, contributing to intelligent livestock management.

## 1 Introduction

Sheep inventory counting, facial recognition, and health analysis are crucial components of daily management on large-scale farms. Accurate counting of sheep in a flock allows for the development of effective breeding plans, which align with animal welfare standards while also reducing farm costs (1). Facial recognition enables precise identification of individual sheep, supporting better tracking and management. The information can be used to tailor feeding plans for each sheep, promoting precise breeding and scientific farm management (2). Additionally, facial expression analysis helps assess the health status of sheep, enabling timely treatment of sick or injured animals and minimizing the risk of disease spread (3). As such, sheep inventory counting, facial recognition, and health analysis hold significant potential for application in precision sheep farming, making them essential tasks for improving farm efficiency and animal welfare (4–6).

Livestock identification methods are generally classified into contact and non-contact types. Contact methods are traditional approaches, including ear markings, ear tags, and radio frequency identification (RFID) (7, 8). Non-contact recognition, on the other hand, typically involves identifying livestock based on physiological characteristics, such as iris and retinal blood vessels (9, 10). Traditional contact methods are limited by distance, and improper installation can cause stress in animals or harm to personnel. Additionally, ear tags and similar methods require manual registration, which is time-consuming and prone to errors. Non-contact recognition, currently reliant on combining retinal and iris data with traditional machine learning, is primarily used for identifying individual animals. However, it does not meet the requirements for identifying sheep or livestock in complex environments (11, 12). Moreover, non-contact methods often involve complicated data collection processes, which are not easily cooperative with livestock, limiting their practical applicability.

In recent years, with the advancement of convolutional neural networks (CNNs), face recognition technology has become increasingly mature. However, studies on sheep recognition remain limited (13, 14). Drawing inspiration from face recognition, several studies have explored the use of CNNs to identify livestock using various biometric features (15–18). Facial recognition technology offers advantages such as being natural, intuitive, and non-contact, eliminating the need for livestock to cooperate with fixed gestures. Additionally, face recognition systems are known for their strong anti-interference capabilities and good scalability. As a result, contactless recognition using visual biometric features has become a promising trend for individual livestock identification. For instance, Xie et al. (19) developed an improved DenseNet-CBAM model for pig face recognition, integrating the Convolutional Block Attention Module (CBAM), which enhanced recognition performance. Wang et al. (20) proposed a multi-scale convolutional neural network-based model for contactless individual pig detection in complex environments, achieving an accuracy of 92%. Li et al. (21) introduced a CNN-based method for detecting pig face feature points, addressing the challenge of accurate feature point detection in livestock recognition. Wang et al. (22) implemented a lightweight pig face recognition model based on a deep convolutional neural network, which demonstrated a high recognition rate in complex environments. Yang et al. (23) applied a YOLOv4-based target detection network that incorporated coordinate information to accurately identify individual cows, with an average recognition accuracy of 93.4%. Traditional cattle identification methods require external tools, which pose safety risks for breeders and may cause physical harm to the cattle. To overcome this, Zhu et al. (24) proposed a bovine face biometric feature extraction method based on image analysis, achieving an accuracy of 95.1%.

The studies mentioned above primarily focus on facial recognition research for pigs and cattle. In contrast, research on sheep facial recognition and expression analysis has made significant progress in the following studies. Xu et al. (25) fine-tuned seven different pre-trained classification network models through transfer learning to assess the effectiveness of existing target classification networks for sheep face recognition, achieving an average detection accuracy of 99.8%. Zhang et al. (26) proposed an improved MobileFaceNet network for sheep face recognition, which achieved 97.91% accuracy in recognizing sheep with small differences, at long distances, and under conditions of low recognition accuracy. Song et al. (27) applied the YOLOv3 recognition network for sheep face recognition, achieving fast and accurate results. Billah et al. (28) introduced a deep learning-based goat facial recognition method, which demonstrated good accuracy, with a recognition rate of 96.4%. For facial expression detection, Han et al. (29) proposed an improved STVGGNet-based algorithm for detecting sheep pain expressions, which achieved an accuracy of 96.06%, addressing the challenges of high experience requirements, low recognition accuracy, high costs, and delays in disease treatment in manual recognition of sheep pain. Noor et al. (30) used transfer learning to fine-tune an existing classification network, achieving effective detection of painful expressions in sheep with accuracies of 96.69% in the validation set and 100% in the test set.

Existing livestock recognition technologies do not integrate individual counting or facial expression analysis functions, which are essential for accurate feeding and real-time health monitoring of individual sheep. To address these limitations, a three-stage system for group sheep recognition and individual multi-part classification is proposed. The system consists of three main steps: multi-part recognition of flock sheep, facial classification, and facial expression analysis. A multi-part recognition network is used to achieve flock counting by targeting the back of the sheep, facial classification is employed for individual sheep identification, and a facial expression analysis network is utilized to assess the health status of the sheep.

## 2 Methods

### 2.1 Production of datasets

#### 2.1.1 Experimental data sources

**Figure 1:**
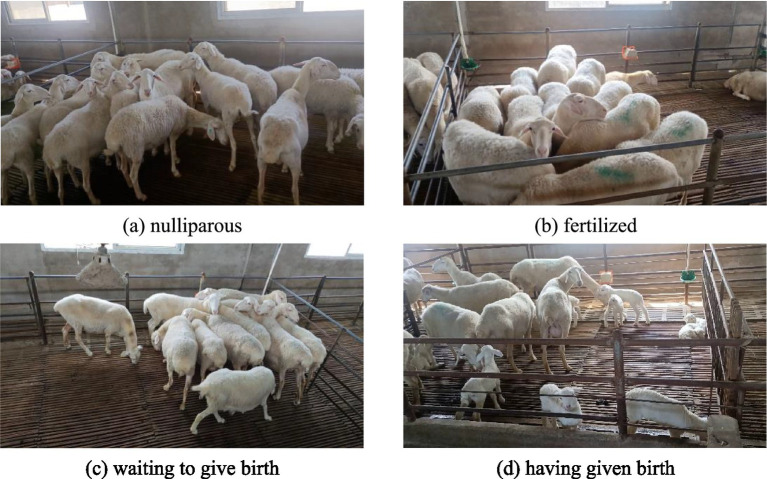
Schematic diagram of the shooting scene. **(A)** Nulliparous. **(B)** Fertilized. **(C)** Waiting to give birth. **(D)** Having given birth.

The video footage of sheep faces used was captured in the standardized indoor sheep farm environment of Luoyang Xiangshun Agricultural and Animal Husbandry Technology Company, located in Luoyang City, Henan Province, China. The test subjects consisted of 30 adult sheep in four different states: nulliparous, fertilized, pregnant, and postpartum. A Canon camera was used for semi-monthly tracking of the sheep in a herd environment, with each video session lasting no less than 30 min. The frame rate was set to 30 fps, and the shooting scenes are shown in Figure 1.

#### 2.1.2 Data similarity processing

The videos were captured at a rate of 25 frames per second, and clear, effective images were selected as sample data. To further filter out similar images and avoid overfitting of the model due to similarity, the perceptual hash algorithm (d-Hash) was used to eliminate the similar images. Both images were resized to 8 × 9 × 3 pixels and then converted to grayscale. The pixel values of each row were compared sequentially: if the former value was larger than the latter, a difference value of 1 was assigned; otherwise, 0 was assigned this process generated two binary 8 × 8 difference matrices. The four characters before and after each row of the difference matrix were converted to hexadecimal, forming two 16-character hash strings. The Hamming distance between the images was then calculated using the XOR method. Only images with a Hamming distance greater than or equal to 12 were retained to eliminate interference from similar images. As a result, a total of 3,078 effective and clear images were selected.

#### 2.1.3 Data amplification and annotation

**Figure 2:**
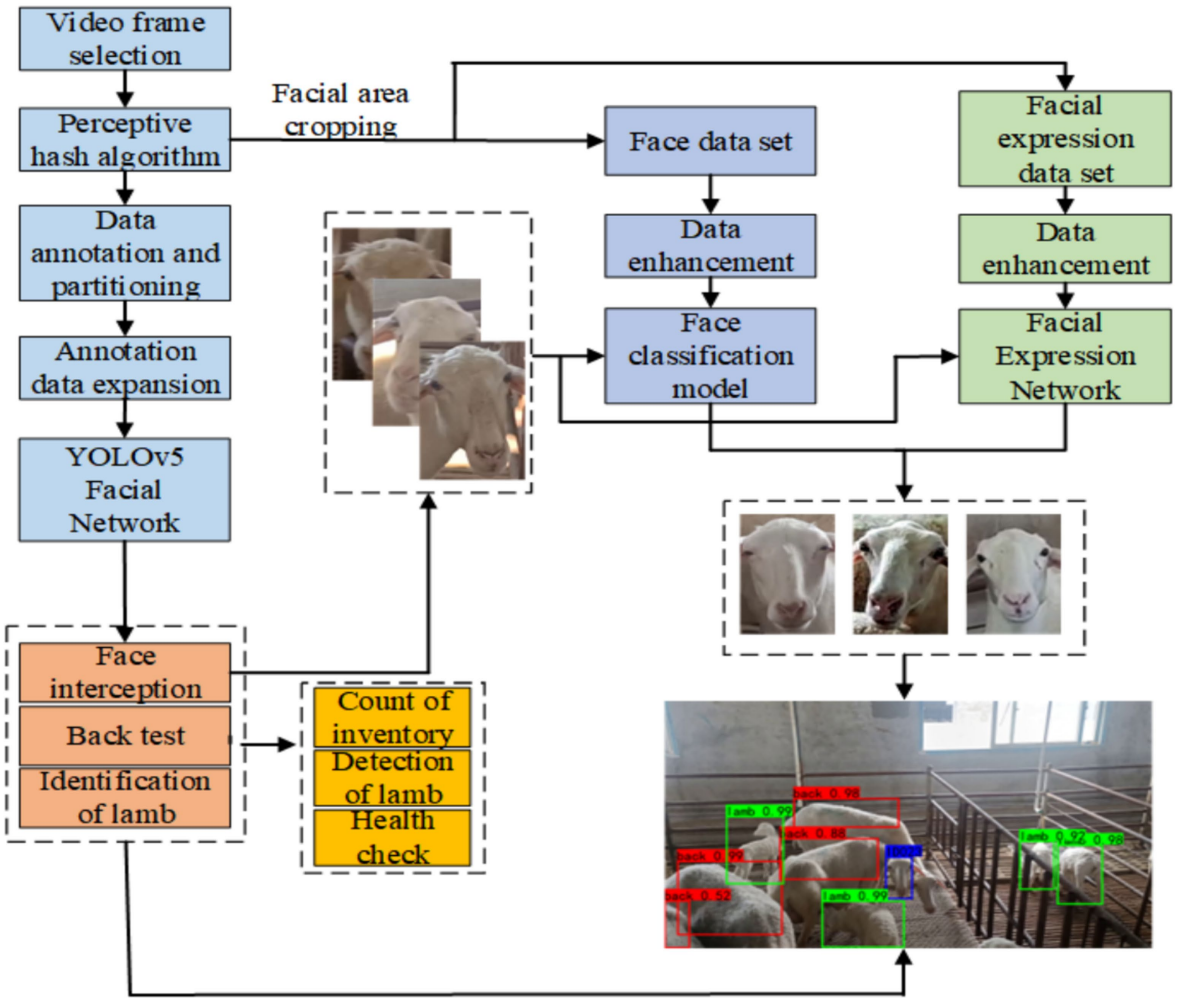
Overall flow chart of facial identity recognition and expression analysis system.

The sheep face recognition network consisted of three parts: a multi-site recognition network for individual flock sheep, a facial classification network, and a facial expression classification network. Three separate datasets—Dataset A, Dataset B, and Dataset K—were used to train the networks, with the overall processing flow shown in Figure 2.

**Figure 3:**
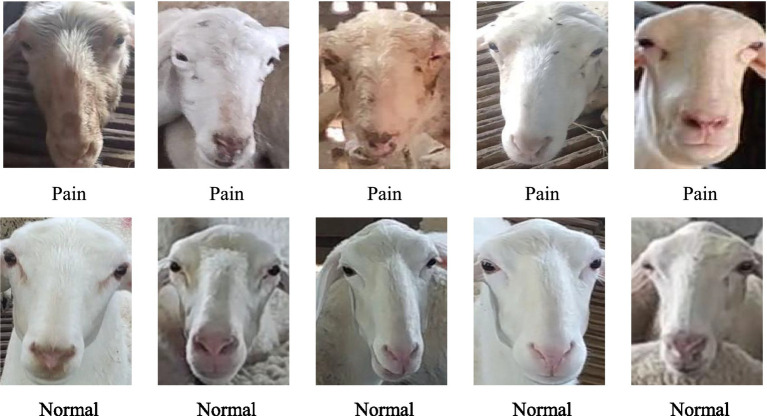
Facial images of sheep in pain and normal states.

Dataset A contained 3,078 pre-screened images. The annotation tool LabelImage was used to annotate the images according to the Pascal VOC format, generating an annotation file in .xml format. Dataset A was then split in a 2:1 ratio. Dataset B consisted of 30 sheep, each with 90 face images, totaling 2,700 images. This dataset was divided in a 2:1 ratio between the training and test sets. Dataset K included two categories of images: healthy and sick sheep, with specific differences between the two categories shown in Figure 3. The healthy images were taken from 15 adult sheep in Dataset B, while the sick images were sourced from an additional five sheep identified as diseased by a local veterinarian.

To enhance the generalization performance of the facial identity and expression analysis system, Datasets A, B, and K were augmented after splitting. Dataset A was augmented using four methods: random rotation (−15° to 15°), horizontal flip, MixUp, and brightness change. The corresponding annotation files were transformed accordingly, generating Training Set C (10,260 images) and Test Set D (5,130 images). For Dataset B, augmentation was performed using random rotation and brightness change, resulting in Training Set E (5,400 frames) and Test Set F (2,700 frames). Dataset K was augmented in the same way as Dataset B, creating Training Set M (2,400 frames) and Test Set N (1,200 frames).

### 2.2 Multi-part identification network for individual flock sheep

#### 2.2.1 Improved face detection model for YOLOv5 sheep

Face and back recognition were used to identify individual sheep and lambs, enabling face interception and sheep counting functions. YOLOv5s was chosen for target detection due to its small model size, fast detection speed, and ease of deployment on mobile devices. YOLOv5s was applied to detect faces, backs, and lamb targets. However, the large number of sheep often led to issues such as stacking and partial occlusion in the large-scale breeding environment, posing significant challenges for the YOLOv5s sheep individual multi-part recognition network. Additionally, the sheep’s body texture features were relatively uniform, with most being pure white, which made it difficult for the YOLOv5s network to distinguish between different parts of the sheep. To address these challenges, improvements were made to the neck and head networks of YOLOv5 to enhance detection performance in obstructed environments and improve the accuracy of sheep individual counting.

A depth factor of *d = 0.33* was applied to the YOLOv5s backbone network to scale down the main architecture of CSPDarkNet53, reducing the number of model parameters and creating a lighter network. A width factor of *w=0.5* was used to decrease the input image size, reducing computational effort and improving detection speed for the multi-part recognition network of individual flock sheep. However, this adjustment also affected the detection performance for small, medium, and large targets, making it less suitable for multi-target detection in complex group sheep environments. Due to variations in sheep body shapes, occlusion, and the different sizes of individuals, especially in the farrowing room where lambs are often obscured by ewes, the model faced significant challenges in detecting sheep in such conditions.

**Figure 4:**
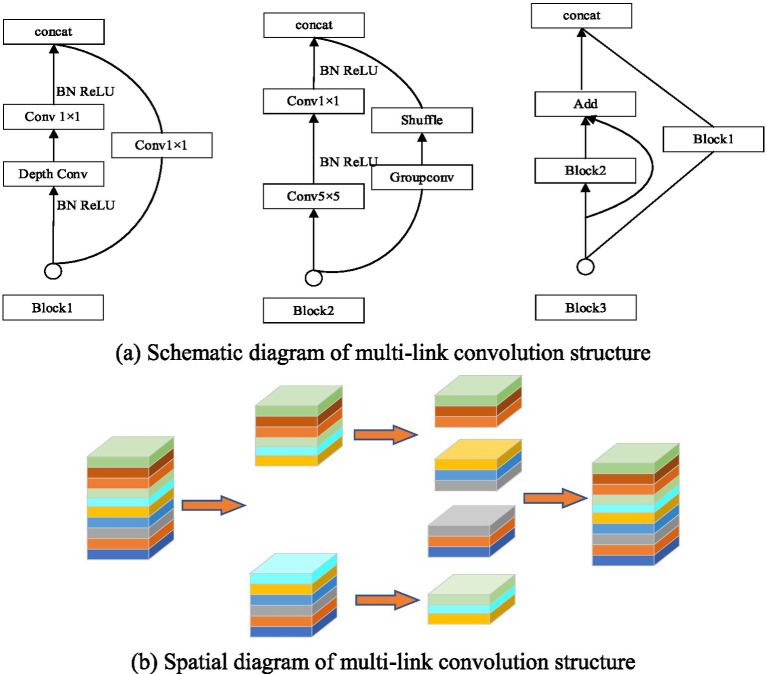
Multi-link convolution blocks. **(A)** Schematic diagram of multi-link convolution structure. **(B)** Spatial diagram of multi-link convolution structure.

To address these issues in complex breeding environments, it was essential to enhance the target detection algorithm’s ability to screen fine-grained features across multiple scales, ensuring effective detection of both large and small objects. A multi-link convolution feature fusion structure was proposed, shown in Figure 4, capable of refining features for small, medium, and large targets. The input feature map was divided into multiple sub-blocks, improving the detection of fine features. Convolution kernels of sizes 1, 3, and 5, along with various convolution types (separable, normal, and group convolution), were applied in specific blocks to enhance feature fusion. The introduction of multi-link convolution reduced the feature fusion path in YOLOv5s, improving the model’s ability to extract higher-order semantic features of sheep while preserving shallow coordinate information. This change allowed for better feature transfer and enhanced recognition of herd sheep. Additionally, multi-link convolution reduced the network model’s parameters by dividing the feature map into smaller blocks, thereby improving detection accuracy and speed for each sheep and lamb part.

The Repconv convolution structure was introduced before the head network to improve the detection effect of different sizes of the group sheep’s face, back and young under complex environment. Repconv contained two different convolution structures of 1 × 1 and 3 × 3. 1 × 1 convolution can further enhance the attention to small target features, and 3 × 3 convolution kernel can enhance the information fusion of high and low order features. Compared with a single common convolution structure, Repconv had a more diversified screening effect on target details to improve the detection effect on targets of different sizes.

**Figure 5:**
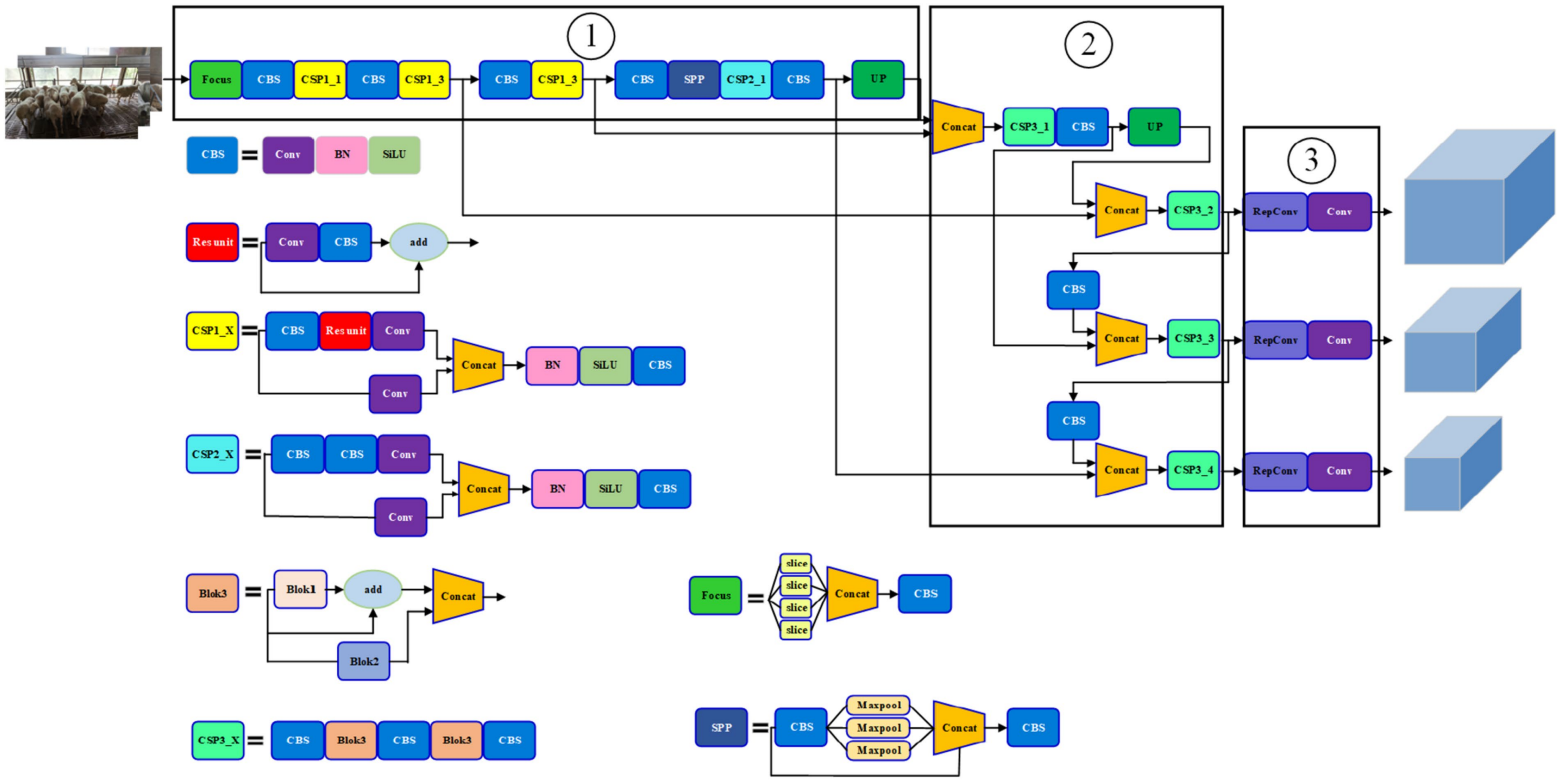
The improved YOLOv5s multi-part recognition network.

Note: CSPn_X stands for Cross stage partial structure, Conv stands for convolutional, BN stands for Batch Norm, CBL stands for Conv+Batch BN + SiLU activation function synthesis module, ResUnit stands for the residual connection module, Concat stands for the feature concatenation operation, UP stands for upsampling operation and Maxpool stands for the pooling operation. Block1, Block2 and Block3, respectively, represent the improved combination structure. Focus represents the focusing module, and slice represents the corresponding slicing operation.

The multi-part recognition network of the improved YOLOv5s sheep is shown in Figure 5. It was easy to see that the network was divided into three parts: ① the CSPDarkNet53 trunk network structure after the same scale; ② the improved path aggregation network (PANet) and ③ the Head network structure.

#### 2.2.2 Anchor frame re-clustering

The anchor frame sizes in the YOLOv5s target detection network are based on clustering from the COCO dataset, which includes 80 categories. Since the anchor frame sizes vary across categories, they cannot be directly applied to sheep face detection. The multi-part recognition network for sheep in this work primarily identifies the face, back, and lamb, each with different shapes and sizes. To improve detection across large, medium, and small targets, the k-means clustering algorithm was used to re-cluster the sizes in the annotation files. This process resulted in 9 anchor frame sizes with different length-to-width ratios: (29, 68), (67, 38), (50, 77), (46, 117), (78, 73), (104, 56), (74, 114), (79, 187), and (142, 115).

#### 2.2.3 Multi-part identification network loss function

YOLOV5s target loss function consists of four parts: positive sample coordinate loss, positive sample confidence loss, negative sample confidence loss and positive sample classification loss. The loss function is calculated as shown in Equation 1. Where $\lambda_{\text{coord}}$ and $\lambda_{\text{noobj}}$ respectively represent positive sample weight coefficients and negative sample coefficients; $\sum_{i=0}^{K\times K}\sum_{j=0}^{M}$ represents traversal all prediction boxes; $I_{ij}^{obj}$ and $I_{ij}^{\text{noobj}}$ respectively represent whether there is an object, that is, there is an object is 1, and there is no object is 0; ${\overset{⌢}{C}}_{i}$ and $C_{i}$ respectively represent the predicted and true values of the samples; $p_{i}$ represents the predicted probability for a certain class; Complete Intersection of Union loss (CIOU) represents the loss function used between the prediction frame and the true as shown in Equation 2; $\rho^{2}\left(b-b^{gt}\right)$ represents the diagonal distance of the minimum closure region between the prediction frame and the true frame; α is used to measure the consistency parameter between the predicted frame and the real frame, and v represents a trade-off parameter as shown in Equation 3.


(1) $$\begin{array}{l} \text{Loss}1=\lambda_{\text{coord}}\sum_{i=0}^{K\times K}\sum_{j=0}^{M}I_{ij}^{obj}\left(2-w_{i}\times h_{i}\right)\left(1-\text{CIOU}\right)\\ -\sum_{i=0}^{K\times K}\sum_{j=0}^{M}I_{ij}^{obj}\left[{\overset{⌢}{C}}_{i}\log\left(C_{i}\right)+\left(1-{\overset{⌢}{C}}_{i}\right)\log\left(1-C_{i}\right)\right]\\ -\lambda_{\text{noobj}}\sum_{i=0}^{K\times K}\sum_{j=0}^{M}I_{ij}^{\text{noobj}}\\ \left[{\overset{⌢}{C}}_{i}\log\left(C_{i}\right)+\left(1-{\overset{⌢}{C}}_{i}\right)\log\left(1-C_{i}\right)\right]-\\ \sum_{i=0}^{K\times K}\sum_{j=0}^{M}I_{ij}^{obj}\sum_{c\in \text{classes}}\left[{\overset{⌢}{p}}_{i}\log\left(p_{i}\left(c\right)\right)+\left(1-p_{i}\left(c\right)\right)\right]p \end{array}$$

(2) $$\text{CIOU}=1-IOU+\frac{\rho^{2}\left(b-b^{gt}\right)}{c^{2}}+\alpha v$$

(3) $$v=\frac{4}{\pi^{2}}{\left(\arctan\frac{w^{gt}}{h^{gt}}-\arctan\frac{w}{h}\right)}^{2}$$

### 2.3 Facial classification network of sheep

Aiming at the phenomenon of feature redundancy in feature extraction networks, GhostNet proposed a lightweight network model, which used linear operations instead of partial convolution to generate a large number of redundant feature graphs to reduce the amount of network computation and improve the speed of the model. GhostNet network was composed of a series of Ghost stream modules stacked. Ghost vector was mainly composed of two structures, separable convolution structure (DWConv) and Ghost module, to achieve the effect of reducing the number of model parameters and improving the detection rate of the model.

#### 2.3.1 Improved GhostNet facial distribution network

**Figure 6:**
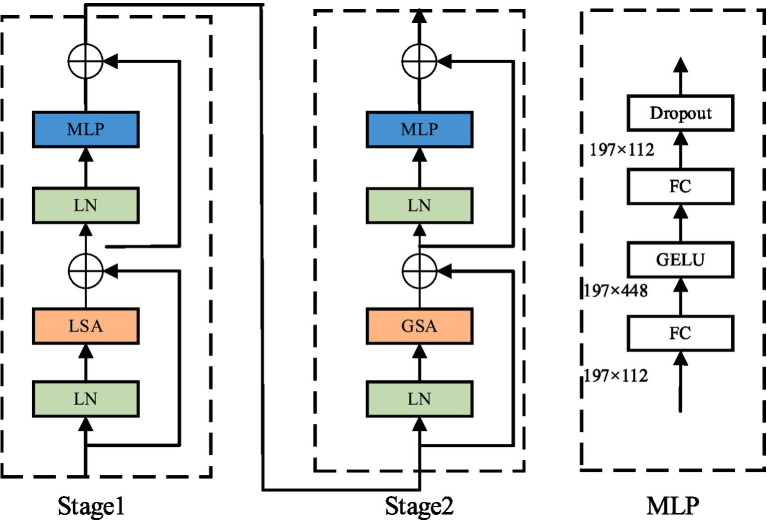
Structure diagram of separable self-attention mechanism.

Although GhostNet feature network generated efficient feature graphs through simple linear feature mapping, its separable convolution structure reduced the information interaction between different feature graphs and may neglect the extraction of key features. In view of the similar facial texture of sheep, small differences in intra-class features, and difficult to distinguish fine grained features, the four-layer spatially separable self-attention mechanism (SSSA) was adopted to effectively replace the sixth stage network structure in GhostNet, so as to improve the extraction of important features in the facial region. The SSSA consisted of the local self-attention mechanism (LSA) and the global subsampled attention mechanism (GSA), the structure is shown in Figure 6. The LSA module captures fine-grained features and short-range information with a window size of 7 × 7, while the GSA processes long-distance and global information, enabling fusion of local features and information exchange between different regions. The fine features of sheep face can be screened and the detection effect of facial classification network can be improved using the alternating link of LSA and GSA.

#### 2.3.2 Facial classification network loss function

CrossEntropy Loss function was used for regression training of sheep face classification network in the improved face classification network, as shown in Equation 4, $y_{j}$ represents the unique thermal coding form corresponding to the real category, and $o_{j}$ represents the probability that the network predicts a certain category.


(4) $$\text{Loss}2=\sum_{j=1}^{q}y_{j}\log\sum_{j=1}^{q}\exp\left(o_{j}\right)-\sum_{j=1}^{q}y_{j}o_{j}$$

### 2.4 Expression analysis network of sheep face

The sheep facial expression analysis network is based on the EfficientNet detection framework, which consists primarily of MBConv convolution blocks. These blocks come in 3 × 3 and 5 × 5 modes, each incorporating three components: standard convolution, separable convolution, and a channel management attention mechanism. After the facial images of sheep are captured, issues such as low resolution and blurred facial expression features often arise, making it challenging for deep networks to extract and train important features. Additionally, the deep layers of the EfficientNet model and high input resolution lead to unnecessary computation, affecting the model’s detection speed. To address these issues, the EfficientNet model is scaled using depth scaling factor *d* and resolution scaling factor *r*. The model was compressed with scaling factors of 1.0, 0.5, and 0.25, reducing the number of parameters, improving detection accuracy, and enhancing the speed of the sheep recognition and classification system.

#### 2.4.1 Facial classification network loss function

Focal Loss function was used to classify sheep faces and regression training sheep facial expressions in the facial expression analysis network as shown in Equation 5. $P_{t}$ represents a probability factor as shown in Equation 6. When the prediction category is consistent with the real category, $P_{t}$ is equal to the probability value of correct prediction, and conversely, the prediction probability value is inverseed; γ represents the adjustable focusing parameter.


(5) $$\text{Loss}3=-{\left(1-P_{t}\right)}^{\gamma }\log\left(P_{t}\right)$$

(6) $$P_{t}=\{\begin{array}{c} p\;\text{if}\;y=1\\ 1-p\;\text{otherwise} \end{array}$$

### 2.5 Model training

#### 2.5.1 Model training and parameter configuration

The facial identity recognition and expression classification system was developed using the PyTorch framework. The system consists of three networks, each trained separately before being integrated for sheep facial identity recognition, individual counting, and facial expression analysis. Transfer learning was applied to enhance the generalization ability and accelerate convergence of the system.

For the improved YOLOv5s multi-part recognition network, pre-training was conducted using the COCO dataset, with the network weights serving as the initialization. This process improves detection accuracy for sheep faces, backs, and lambs in a complex environment. The training settings include an image size of 640 × 640, a batch size of 16, and 300 epochs. After each epoch, the system automatically saves the best-performing weights. The backbone network is frozen for the first 50 epochs, with an initial learning rate (LR) set to 0.001, which is reduced to 0.0001 for the next 250 epochs. The LR is smoothed using the cosine annealing algorithm to enhance feature extraction in the facial recognition network.

Regarding the sheep facial classification and expression analysis networks, the Mini-ImageNet dataset was used for transfer learning. The input image size was set to 224 × 224, and the LambdaLR scheduling strategy was employed to adjust the LR periodically. The initial LR was set to 0.01 for both sub-networks, with the SGD optimizer used to optimize the model parameters.

#### 2.5.2 Model evaluation index

To comprehensively evaluate the network detection performance of the facial identity recognition and expression classification system, several evaluation metrics are employed, including average precision (AP), mean average precision (mAP), accuracy, precision, recall, frames per second (FPS), model weight size (Weight), model parameter count (Params), model computation (FLOPS), and memory usage during model inference (Memory). FPS refers to the number of images processed per second. AP is calculated by plotting the precision-recall (P-R) curve, with recall on the horizontal axis and precision on the vertical axis, and integrating to find the area under the curve. mAP is the mean of the AP values across all categories. The calculation formulas for these metrics are as follows:


(7) $$\text{Precision}=\frac{TP}{TP+FP}$$

(8) $$\text{Recall}=\frac{TP}{TP+FN}$$

(9) $$\text{Accuracy}=\frac{TP+TN}{TP+TN+FP+FN}$$

(10) $$AP=\int_{0}^{1}\text{Precision}\cdot \text{Recall}$$

(11) $$\text{mAP}=\frac{\sum_{i=1}^{N}AP_{i}}{N}$$

In Equations 7–11, TP represents the number of positive samples predicted by the model that are consistent with the real label; FP represents the number of samples predicted by the model that are inconsistent with the actual positive samples; FN (False Negative) represents the number of samples predicted by the model that are inconsistent with the actual negative samples; TN represents the number of samples predicted by the model that are consistent with the actual negative samples.

## 3 Results and analysis

### 3.1 Comparison of the results of multi-part recognition model of sheep individual

#### 3.1.1 Comparison of the results of different types of recognition algorithms

The same dataset C was used to train common target detection algorithms, such as the YOLO series, SSD, Faster-RCNN, CenterNet, and EfficientDet, to comprehensively evaluate the performance of the improved individual multi-part identification network. These detection algorithms were tested using test set D from the multi-part identification data of sheep individuals. The network models were assessed across six aspects: FPS, weight, mAP, Params, FLOPs, and memory usage. The test results are presented in Table 1. The mAP values in Table 1 were all obtained with an IOU threshold of 0.5.

**Table 1:** Comparison of the results of different models.

Model	Fps/s	mAP/%	Weight/M	Params	Flops/G	Memory/M
SSD	83	81.01%	91.6	26,151,824	31.39	206.92
CenterNet	74	92.60%	124.0	32,665,432	22.15	610.50
Faster-RCNN	18	91.88%	521.0	28,469,983	461.65	532.34
EfficientDet	19	85.85%	15.0	3,839,060	2.12	306.24
YOLOv4-Tiny	129	77.12%	22.4	5,918,006	3.43	72.94
YOLOv4	34	92.74%	244	64,040,001	29.95	606.54
YOLOX-Nano	50	88.09%	3.7	900,459	1.24	230.00
YOLOX-Tiny	64	92.91%	19.4	5,038,395	7.59	260.22
YOLOX-s	61	93.40%	34.3	8,945,035	13.34	346.70
YOLOX-m	46	94.59%	96.8	25,291,755	36.78	646.21
YOLOX-l	29	94.32%	207.0	54,162,635	77.70	1030.10
YOLOX-x	18	94.01%	378.0	99,013,675	140.8	1498.36
YOLOv5-s	64	93.29%	27.1	711,785	8.27	286.78
YOLOv5-m	46	94.17%	80.6	21,133,185	25.33	555.53
YOLOv5-l	29	95.83%	178.0	46,733,665	57.28	908.65
YOLOv5-x	18	95.98%	378.0	87,257,832	108.68	1344.52

As shown in Table 1, the highest FPS of YOLOv4-tiny is 129, making it the fastest in terms of detection speed; however, its mAP is only 77.12%, indicating low detection accuracy. YOLOv5-x achieves the highest detection accuracy with an mAP of 95.98%, but its weight, parameters, and FLOPs are too large, making it less suitable for lightweight model applications. YOLOv5-s has an FPS of 64, mAP of 93.29%, weight of 27.1, parameters of 711,785, and FLOPs of 8.27, offering a good balance between detection accuracy and speed. Therefore, YOLOv5-s is chosen as the baseline model for the multi-part recognition network.

#### 3.1.2 Comparison of the results of improved multi-part recognition models of sheep individual

Improvements were made to YOLOv5s and experiments were conducted, as shown in Table 2. YOLOv5s-MCFB refers to the multi-part recognition network for sheep individuals that incorporates multi-link convolution feature fusion blocks into the path aggregation network, while YOLOv5s-MCFB+ further integrates the Repconv convolution structure on top of the multi-link convolution feature fusion blocks.

**Table 2:** Comparison of the test results of the improved multi-part identification models of sheep individual.

Model	Fps/s	mAP/%	Weight/M	Params	Flops/G	Memory/M
YOLOv5s	64	93.29	27.1	711,785	8.27	286.78
YOLOv5s-MCFB	57	95.77	59.7	14,522,120	15.96	485.93
YOLOv5s-MCFB+	53	95.84	73.9	18,580,104	18.12	425.77

According to Table 2, the mAP for YOLOv5s-MCFB and YOLOv5s-MCFB+ reaches 95.77 and 95.84%, respectively. The FPS of these models is slightly lower than that of YOLOv5s, at 57 FPS and 53 FPS, respectively. Compared to YOLOX Nano, YOLOX Tiny, YOLOX-s, YOLO-m, YOLOX-1, YOLOX-x, YOLOv5s, YOLOv5m, and YOLOv5l, the mAP of YOLOv5s-MCFB+ increased by 7.75, 2.93, 2.44, 1.25, 1.52, 1.83, 2.55, 1.67, and 0.01%, respectively. This demonstrates the effectiveness of the improvements proposed in this paper.

To evaluate the detection performance of the improved multi-part recognition network for individual sheep counting, the backs, faces, and lambs in 120 images were counted. As shown in Table 3, a total of 187 faces, 936 backs, and 185 lambs were identified, with 35 individuals experiencing severe occlusion, bringing the total to 1,173 sheep. YOLOv5s-MCFB+ achieved the highest detection accuracy in counting sheep individuals, detecting 1,161 individuals with only 12 missed detections, resulting in a detection accuracy of 98.97%. When compared with YOLOv5s, YOLOv5m, YOLOv5l, and YOLOv5x models, the results showed increases of 5.2, 3.32, 2.13, and 0.68%, respectively.

**Table 3:** Comparison of individual counting between the improved multi-part recognition model and the YOLOv5 series models.

Model	Face	Back	Lamb	Total	Shelter	Predict	Omission	Accuracy/%
YOLOv5s	187	936	185	1,138	35	1,100	73	93.77
YOLOv5m	187	936	185	1,138	35	1,122	51	95.65
YOLOv5l	187	936	185	1,138	35	1,136	37	96.84
YOLOv5x	187	936	185	1,138	35	1,142	31	97.35
YOLOv5s-MCCB	187	936	185	1,138	35	1,153	20	98.29
YOLOv5s-MCCB+	187	936	185	1,138	35	1,161	12	98.97

**Figure 7:**
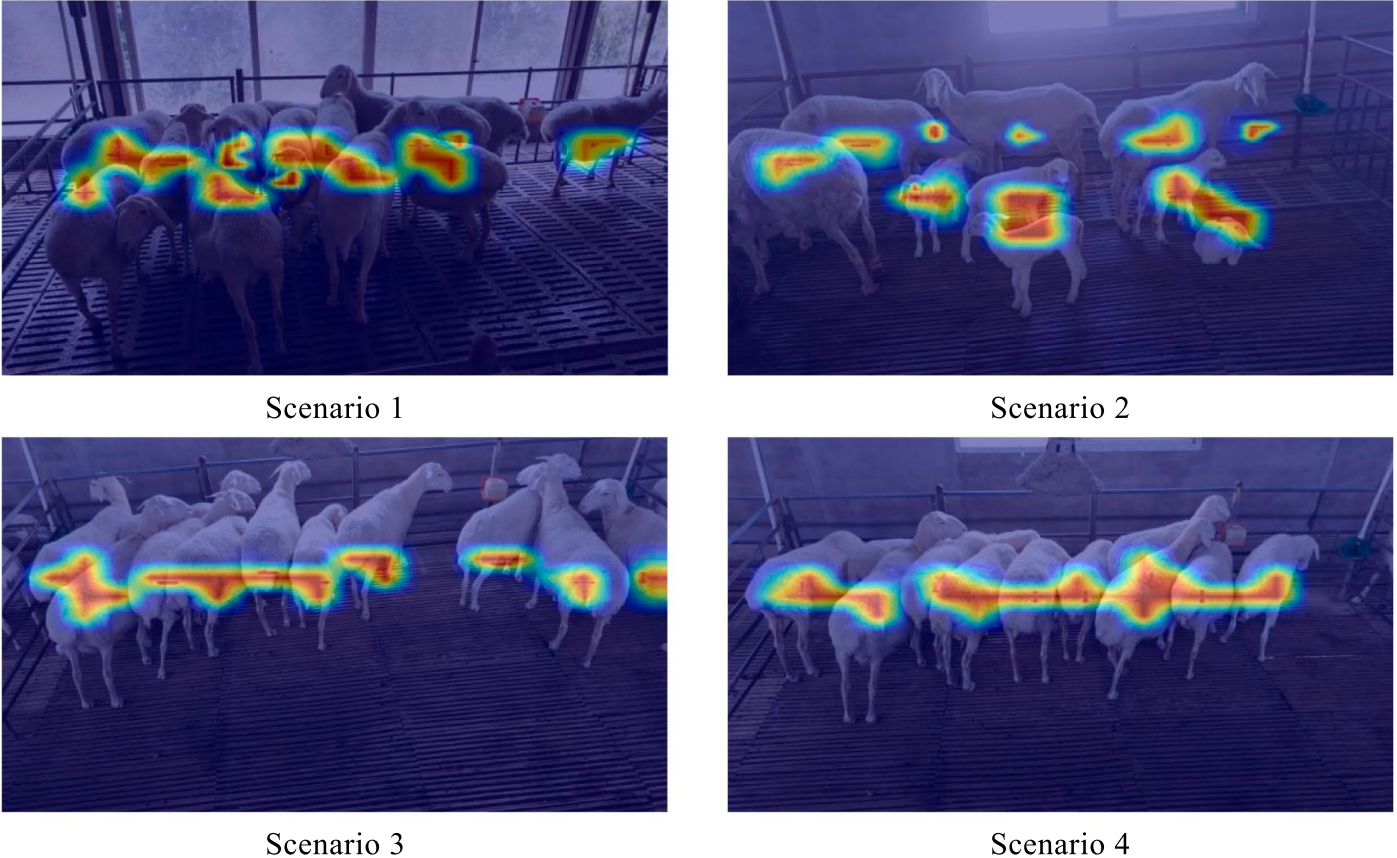
Class activation heat map of different sheep parts in the improved multi-part recognition network.

Since discussed in Figure 7, the prediction outcomes of the YOLOv5s-MCFB+ multi-part recognition model were visualized using class activation heat maps in order to further confirm the efficacy of the enhanced multi-part recognition network. The areas that the model concentrated on throughout the identification phase are indicated by these heat maps. It is clear that the model focuses more on the back and lamb aspects of the sheep and less on the face, which explains why the model is more successful at identifying the back and lamb than the face.

### 3.2 Comparison of the results of the improved facial classification network model

ResNet50, MobileNetV2, MobileNetV3, MobileVit, GhostNet and the improved facial classification network model were used for comparative training of sheep face classification dataset E. The performance of these networks was tested using test set F, and the results are summarized in Table 4. MobileNetVit, MobileNetVitxs, and MobileNetVitxxs represent different versions of the same classification network. GhostNetVits, GhostNetVitm, and GhostNetVitl correspond to networks in which varying amounts (1, 2, 3) of LSA-GSA three-stage face classification networks were introduced into the sixth-stage structure of the GhostNet classification network. The Multi-Head Attention mechanism, which integrates different network modules for deeper feature extraction, was also explored. Specifically, GhostNetVitm2, GhostNetVitm, and GhostNetVitm8 represent the variations of the network structure with 2, 4, and 8 Multi-Head Attention layers, respectively.

**Table 4:** Results comparison between the improved sheep face classification model and other models.

Model	Precision	Recall	Fps/s	Train_acc	Test_acc	Weight/M
ResNet50	99.4%	99.4%	47	96.5%	98.3%	81.3
MobileNetV2	96.2%	93.8%	76	94.3%	94.9%	8.86
MobileNetV3	96.5%	96.2%	66	94.7%	95.2%	16.3
MobileNetVit	92.8%	92.7%	52	90.7%	91.3%	19.2
MobileNetVitxs	95.8%	95.3%	55	93.4%	94.3%	7.81
MobileNetVitxxs	96.1%	96.3%	66	94.5%	94.7%	4.11
GhostNet	97.4%	96.3%	71	95.3%	95.8%	15.2
GhostNetVits	88.3%	84.1%	66	90.1%	89.5%	12.7
GhostNetVitm2	99.3%	99.4%	58	96.5%	98.1%	19.1
GhostNetVitm	99.4%	99.6%	58	96.7%	98.9%	19.1
GhostNetVitm8	97.4%	96.0%	58	95.4%	96.2%	19.1
GhostNetVitl	99.3%	99.2%	47	95.8%	98.2%	21.0

As shown in Table 4, the Precision, Recall, and Accuracy of the GhostNetVitm on the training and testing datasets are 99.4, 99.6, 96.7, and 98.7%, respectively, outperforming GhostNet, GhostNetVits, and GhostNetVitl network. This indicates that adding the two-layer LSA-GSA structure to the GhostNet network yields the best results. Comparing different numbers of multi-head attention mechanisms, the results show that adding the four-layer multi-head attention mechanism yields the best performance. Therefore, the GhostNetVitm network is chosen for facial identity recognition of sheep.

To verify the effectiveness of the improved face classification network, the loss change curves for GhostNet, GhostNetVits, GhostNetVitm2, GhostNetVitm, GhostNetVitm8, and GhostNetVitl on test set F are plotted, as shown in Figure 7.

**Figure 8:**
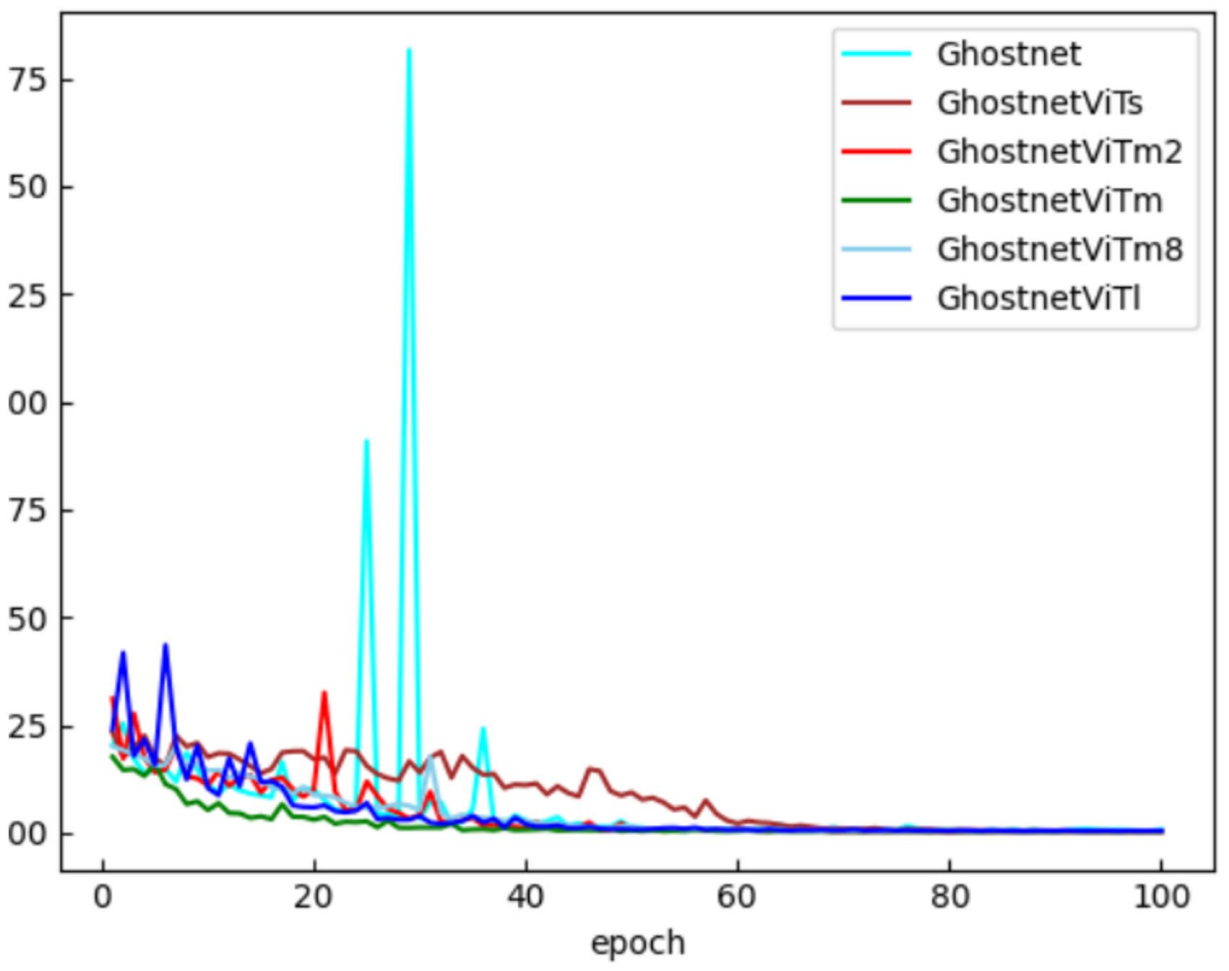
Loss curve of the improved face classification network ablation test.

As observed in Figure 8, the loss curve for the GhostNet facial recognition model exhibits significant fluctuations and a slow convergence rate. In contrast, the loss curve for GhostNetVits fluctuates more gently, although the overall loss value is slightly higher than that of the other networks. The loss curves for GhostNetVitm2, GhostNetVitm, GhostNetVitm8, and GhostNetVitl show minimal differences. Among these, the improved GhostNetVitm face classification network achieves the lowest loss value and the fastest convergence, further demonstrating the effectiveness of the improved sheep face classification network.

**Figure 9:**
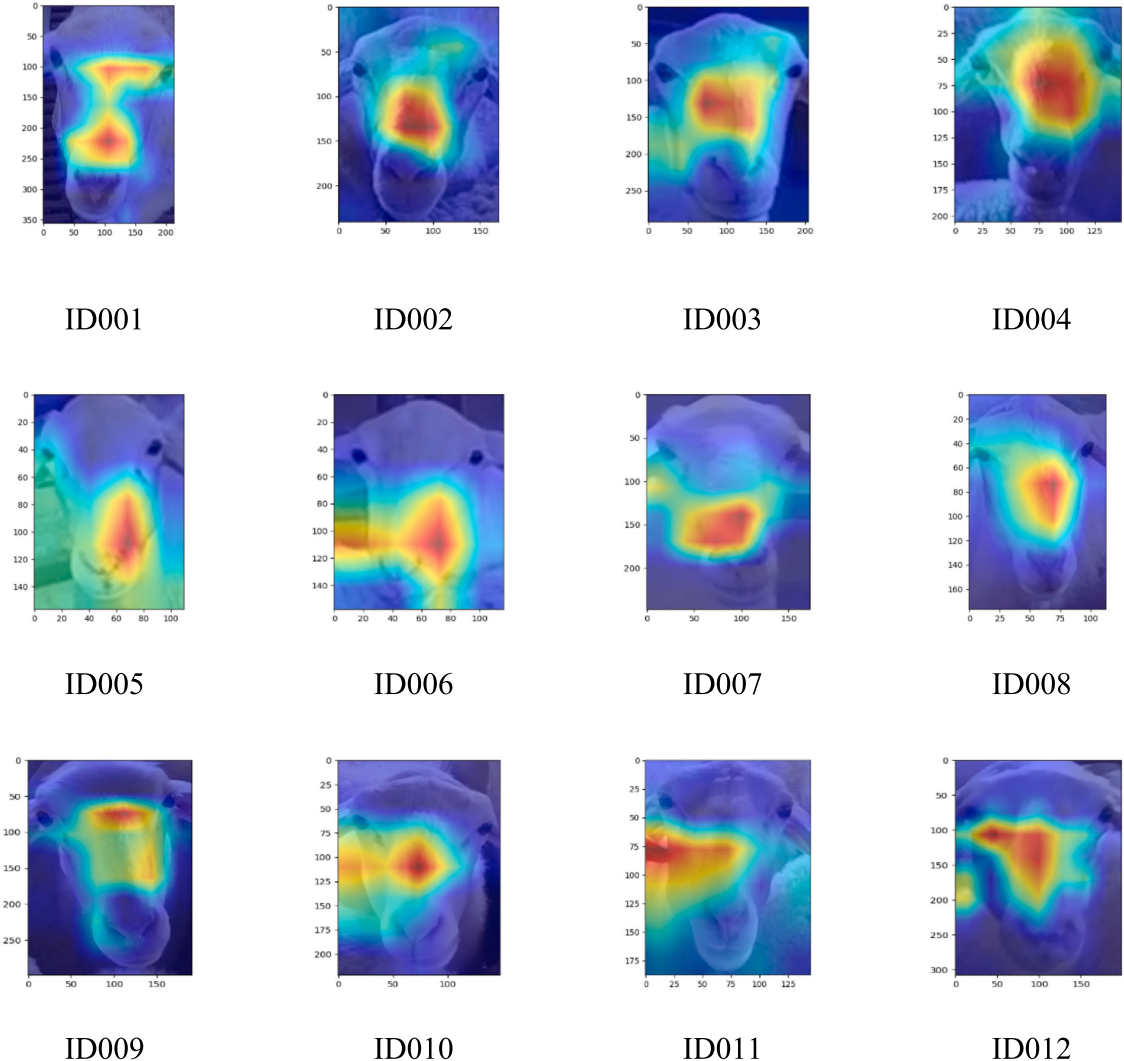
Improved facial classification network class activation heat map.

Additionally, to demonstrate that the improved face classification network model focuses on the features of different facial regions of sheep, 12 facial images from ID001 to ID012 were selected. The class activation heat map was applied to visualize the last layer of the convolutional feature map. The specific results are shown in Figure 9.

The darker regions in Figure 8, which correspond to higher activation areas, indicate that the facial classification network model places more emphasis on the features in those regions. This suggests that these areas play a more significant role in the recognition process of the sheep’s facial identity. The facial features of sheep are primarily focused around the bridge of the nose, with some concentration on the sides of the nose bridge. This highlights that the improved facial classification model primarily differentiates individual sheep based on the nose bridge area. For sheep with less distinct features in the nose bridge region, the model relies more on the sides of the nose bridge for identification.

### 3.3 Comparison of results of improved facial expression network model

In order to show the detection effect of the improved facial expression classification network, the model is trained and verified by using facial pictures of healthy sheep in the natural state and painful expression pictures in the pathological state. EfficientNet0.5 represents scaling of the depth of the network layers and the input image resolution size by 0.5 times, respectively. In Table 5, EfficientNet0.25 represents scaling for the depth of network layers and the resolution size of the input image by 0.25 times, respectively. EfficientNet0.5 facial expression classification network can achieve 99.5, 98.0%, 140Fps/s, 99.2%, and 2.64 M in terms of Precision, Recall, Fps, test set accuracy, and Weight size, respectively. Compared with EfficientNet, it has improved 5.4, 4.4 and 3.6% in Precision, Recall, test set accuracy and other metrics imitations. Compared to EfficientNet0.25, the improvement is 0.6, 2.3 and 0.5%, respectively. In terms of detection frame rate, EfficientNet0.25 has the fastest detection speed, up to 185Fps/s. EfficientNet0.5 has a detection speed of 140Fps/s, which is a significant improvement over EfficientNet and slightly lower than the detection speed of EfficientNet0.25 In terms of weight size, the EfficientNet0.25 model is the smallest, with a weight of only 0.55 M. The weight of EfficientNet0.5 is 2.64 M, which is a substantial reduction compared to EfficientNet.

**Table 5:** Comparison of ablation test results of the improved facial expression classification network.

Model	Precision	Recall	Fps/s	Train_acc	Test_acc	Weight/M
EfficientNet	94.1	93.6	76	93.7	95.6	15.50
EfficientNet_0.5_	99.5	98.0	140	98.2	99.2	2.64
EfficientNet_0.25_	98.9	95.7	185	98.0	98.7	0.55

**Figure 10:**
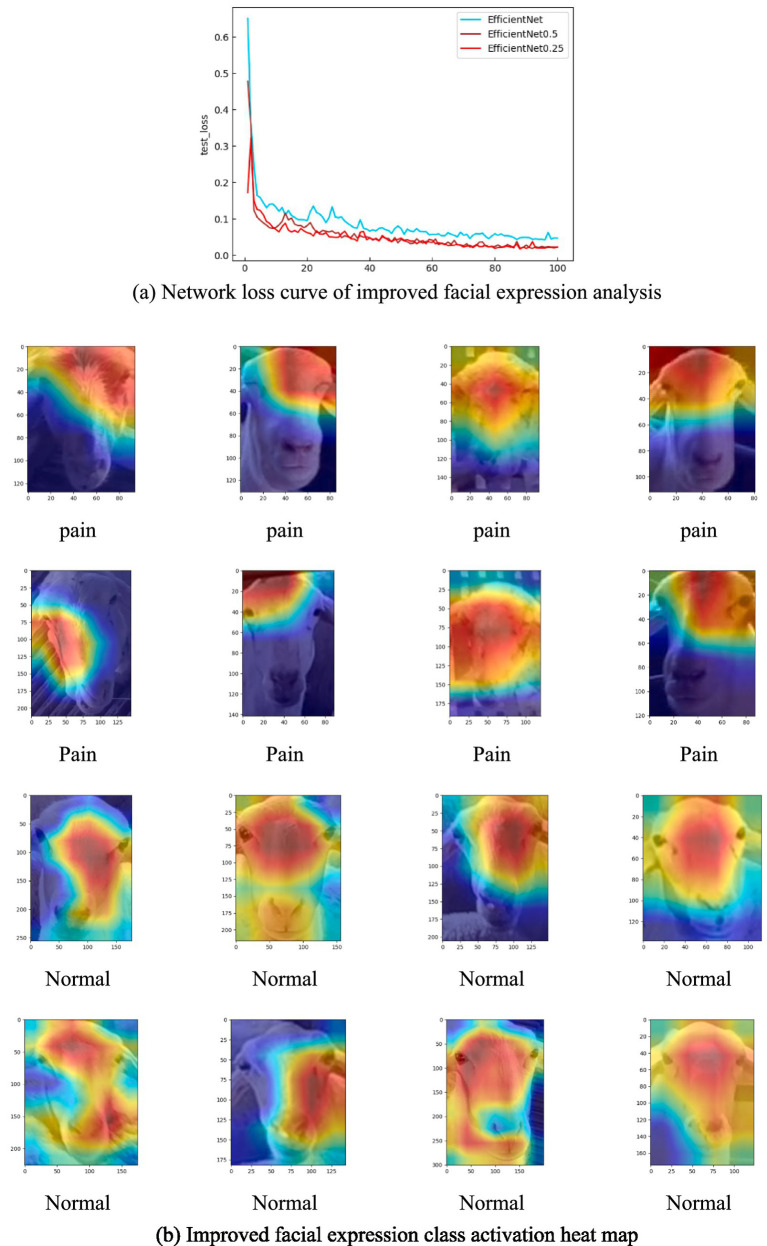
Network loss curve and activation heat map. **(A)** Network loss curve of improved facial expression analysis. **(B)** Improved facial expression class activation heat map.

To further verify the effectiveness of the network improvement, the loss curves of the above three network structures in the test set were plotted in this experiment, as shown in Figure 10A. EfficientNet_0.5_ loss curve has the best smoothness with increasing epoch, and the loss value is the lowest among the three curves. Therefore, for the sheep facial expression analysis network, appropriately reducing the depth of the network and the resolution of the input images helps improve both the detection accuracy and speed. It effectively reduces the risk of losing important features during the feature extraction process, enhancing the robustness of the model. In addition, this study selected facial images of sheep in healthy state and painful images in sick state, a total of 16 images. Heatmap visualization was performed on the facial expression analysis network EfficientNet0.5, as shown in Figure 10B.

The heat map reveals that the improved facial expression classification network primarily focuses on the eye region for sick sheep. In contrast, for facial images of sheep in a healthy state, the network emphasizes the region between the two eyes and the side face area. The distribution of important features in the facial visualizations indicates that the eye region plays a crucial role in distinguishing between the two conditions—healthy and sick sheep. This highlights the eye area as the key feature for identifying the different morphological states of the sheep.

## 4 Discussion

This study focused on the analysis and summary of the detection results of individual multi-part recognition network, facial classification network and facial expression network of sheep. It was found that the improved individual multi-part recognition model focused more on the back and lamb features of sheep, but lacked focus on the facial region of sheep from the results of the improved individual multi-part recognition network. The improved multi-part recognition network for individual flock sheep may be missing when physical, behavioral and side faces are included. However, considering the robustness of the individual multi-part recognition network, the network should selectively discard some images with obviously incomplete faces, so as to reduce the risk of misidentification and improve the accuracy of the facial classification network Guo et al. (31). Therefore, this balancing issue needs to be discussed further. In conclusion, the improved individual multi-part recognition network of sheep needs to be improved the detection effect of face as much as possible in all aspects, as well as back and pup detection performance.

The facial classification network is mainly responsible for judging the individual facial images captured by the multi-part recognition network to realize individual identity recognition in the herd environment. The detection results of the improved facial classification network showed that the network mainly discriminates the nasal bridge region of sheep to achieve the identification of individual sheep. And when individual nasal region features are not obvious, the model needs to make critical distinctions by features on both sides of the nasal bridge. Whether or not individual sheep identity can be reliably determined by features on the nasal bridge is still to be verified in future work, considering whether it is influenced by sheep species and the number of individual sheep Hitelman et al. (32).

For the facial expression analysis network, this study focuses on the differences and connection between the natural expression of healthy sheep and the painful expressions of sick sheep to achieve the initial health management of group sheep. Through the visualization of the training process, it was observed that the eyes of healthy sheep are more lively, while the eyes of sick sheep appear dull Fitzpatrick et al. (33). Therefore, it is feasible to judge the health status of sheep by using the features of sheep eye region in a certain principle.

## 5 Conclusion

(1) For the problems that the target recognition algorithm with large number of network parameters has low detection speed but it has low detection accuracy with small network parameters, an improved multi-part recognition algorithm was proposed based on YOLOv5s as the prototype. A multi-link convolution residual feature fusion structure was introduced into the YOLOv5s path aggregation network structure to improve the screening ability of fine-grained features of objects of different sizes. In order to further improve the detection effect of dense targets and the detection ability of small targets, a layer of Repconv convolution structure was added to the head part on the basis of the introduction of the multi-convolution residual feature fusion structure in the sheep individual multi-part recognition algorithm, so as to realize the detection of small targets in complex environments.
(2) Aiming at the characteristics of sheep with similar facial texture, small intra-class feature variability, and fine-grained features that were not easy to distinguish, improvements were made on the basis of the Ghostnet facial classification network. In order to effectively replace the sixth stage network structure in GhostNet, the four-layer SSSA was used to enhance the feature extraction ability of sheep facial features and improve the detection accuracy of the facial classification network.
(3) For the improvement of the detection speed and effectiveness of the facial expression classification network, model compression tests were performed for the expression analysis network EfficientNet. The test results showed that certain compression EfficientNet helped to improve the model detection accuracy and enhance the model robustness and generalization performance.

## Data Availability

The data supporting the findings of this study are available from the corresponding author upon reasonable request.
